# Efficient purification of flavonoids from bamboo shoot residues of *Phyllostachys edulis* by macroporous resin and their hypoglycemic activity

**DOI:** 10.1016/j.fochx.2022.100505

**Published:** 2022-11-12

**Authors:** Yanbin Wang, Yalan Zhang, Junwen Cheng, Jiancheng Zhao, Rui Shi, Liang He, Qin Li, Yongjian Chen

**Affiliations:** a The Key Laboratory of Biochemical Utilization of Zhejiang Province, Department of Forest Foods, Zhejiang Academy of Forestry, Hangzhou 310023, China; b Bamboo Shoots Engineering Research Center of the State Forestry Bureau, Department of Bamboo, Zhejiang Academy of Forestry, Hangzhou 310023, China; c College of Light Industry and Food Engineering, Nanjing Forestry University, Nanjing 200093, China; d Zhejiang Shengshi Biotechnology Co., Ltd., Huzhou 313399, China

**Keywords:** Bamboo shoots residue, Flavonoids, Enrichment, Adsorption kinetics, Hypoglycemic activity

## Abstract

The adsorption/desorption process of flavonoids obtained from bamboo shoots residues of *Phyllostachys edulis* (RPEFs) was established by resin screening, adsorption isotherms, adsorption kinetics and dynamic tests. Results indicated the Langmuir equation fairly fitted the isotherms data and the pseudo-second-order model accurately described its kinetic adsorption on HPD-500 resin. The optimal process was as follows: 2 mg/mL of solution, 2.8 BV feed volume under 2 BV/h flow rate for adsorption; 5 BV volume of 70 % ethanol with a flow rate of 2 BV/h for desorption. After enrichment, the flavonoids in RPEFs increased from 1.39 % to 37.34 %. The analysis of UPLC-TOF-MS/MS uncovered that the purified RPEFs were composed of four major flavonoids. It had potent hypoglycemic activity *via* PI3K/AKT pathway by upregulating the protein expression of PI3K, AKT, IRS-1, and GLUT4 in a dose-dependent manner based on HepG2-IR cells. Those results provide possible utilization of *Phyllostachys edulis* by-products in food additives.

## Highlights

• HPD-500 resin was suitable for the efficient purification of RPEFs.
• The pseudo-second-order and Langmuir models correlated well with adsorption.
• The purified RPEFs mainly contained four flavone glycosides, especially schaftoside.
• It showed potent hypoglycemic activity via PI3K/AKT pathway in HepG2-IR cells.

## Graphical abstract

**Figure:**
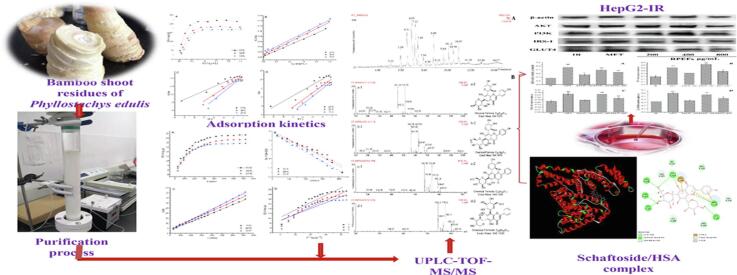

## Introduction

Bamboo shoots of *Phyllostachys edulis*, belonging to the genus Phyllostachys Sieb, are widely distributed in southern China with a yield of beyond 100 million tons per year. Due to their richness in protein, carbohydrate, flavonoids, minerals, and other nutrients, they have attracted great attention on wide consumption as healthy food (Sun et al., 2016, Luo et al., 2008a, Luo et al., 2008b). However, almost 70 percent of them are not utilized effectively and abandoned directly, resulting in tremendous bamboo shoots processing wastes and environmental pollution (Wang et al., 2020, Luo et al., 2008a, Luo et al., 2008b). Fortunately, like other biomass, there are still bioactive compounds left in outer sheaths and basal segments of the bamboo shoot processing by-products (Lin et al., 2018, Li et al., 2013). Among them, the flavonoids from the processing residue of *Phyllostachys edulis* bamboo shoot (RPEFs) have been studied as bioactive substances for the potentials of antioxidant (Hamed et al., 2019), antimicrobial (Cheng et al., 2020) and antidiabetic activities (Luo et al., 2021). Many pieces of evidence have supported that the purified flavonoids could increase health awareness about the consumption of their extracts (Panee, 2015). Accordingly, efficient improvement of this kind of component would boost its application in food additive, drug and cosmetics industries (Ruan et al., 2013).

For the enrichment of flavonoids from natural plants, a number of endeavors have been employed including two aqueous phase extraction (Xie et al., 2015), thin layer chromatography (Zou et al., 2015), preparative HPLC (Guo et al., 2015), high-speed counter-current chromatography (Liang et al., 2013), supercritical fluid purification (Xi et al., 2015), membrane filtration (Zhang et al., 2014) and different packing support column chromatography (Hwang & Chung, 2018). It is not satisfactory that these purification technologies still have drawbacks of excessive cost, longer operating-recycle, and tough industrial enlargement (Belwal et al., 2018). Macroporous adsorption resin as emerging column stuff has been developed to achieve better effects on the purification process, which is a kind of synthetic polymer adsorbent with excellent mechanical property, selective polarity, special pore dimension, and considerable surface area (Du et al., 2012, Limwachiranon et al., 2019). Therefore, it’s welcome to be put into the application of purity enhancement of flavonoids, such as the separation and purification of flavonoids from licorice (Luo et al., 2019), the efficient enrichment of total flavonoids from cabbage (Chen et al., 2022), the purification of total flavonoids from *Gnaphalium affine* D. Don (Li et al., 2021) and the good adsorption of flavonoids from baobab fruit pulp (Ismail et al., 2020). However, little work has been done in-depth on the potential features of flavonoids from the bamboo shoot residues of *Phyllostachys edulis* so far.

This study was conducted with the aim to build a fast, efficient and applicable process for the enrichment of RPEFs and investigate the property of major components for further valorization in industry. For that purpose, the ideal process for RPEFs purification was achieved by selection of different macroporous resins, followed by adsorption isotherms, adsorption kinetics, and eluent concentration selection for the desorption capacity. Moreover, the purified RPEFs were analyzed with UPLC-TOF-MS/MS. Then their hypoglycemic activity was tested based on the insulin resistance model (HepG2-IR).

## Materials and methods

### Materials and reagents

Methanol, formic acid and acetonitrile were of HPLC-grade from Tedia Company Inc. (Ohio, USA). Al(NO_3_)_3_, NaNO_2_, and NaOH were analytically pure, both of which were subscribed from Shanghai Aladdin Bio-Chem Technology Co., Ltd. HPD-100, HPD-500, AB-8, NKA-9, D-101, XAD-4, DM-301, and HP-20 came from Tianjin Nankai Hecheng Co., Ltd. DMEM high glucose medium was ordered from Wuhan Procell Co., Ltd. Glucose detection kit was purchased from Golden Clone (Beijing) Biotechnology Co., Ltd. β-actin, CCK8, RIPA, BCA, FBS and PI3K were obtained from Beyotime Biotechnology Co., Ltd. Insulin, AKT, IRS-1, GLUT4 and all the antibodies were products of Nuoyang Biotechnology Co., Ltd.

### Treatment of raw materials and resins

The outer skins and bottom parts of bamboo shoot residue from *Phyllostachys edulis* were collected from the bamboo and tea plantation in Jingshan Town, Yuhang District, Hangzhou City, Zhejiang Province, which were cut into pieces and washed with distilled water. After oven drying for 24 h at 60℃, the raw materials were subjected to crusher ground for 60 mu and then stored at room temperature.

Eight resins (HPD-500, HPD-400, HPD-100, D101, DM301, HP-20, AB-8, HP-20, and NKA-9) were immersed overnight in 95 % ethanol and flushed with ultrapure water to be ethanol-free. Then they were subjected to rinse with 2 BV 5 % HCl and 5 % NaOH solution successively, prior to the elution with ultrapure water to make pH value-neutral. Finally, the treated resin was oven-dried under 60 °C before use. (Chen et al., 2022).

### Extraction of crude RPEFs

The crude RPEFs was isolated following the reference made by Hou et al (2020) with some modification. 10.00 g of bamboo shoots residue powder was transferred into a flask, and added with 250 mL of 55 % ethanol (solid–liquid ratio 1:25). The mixture was maintained in the hot water at 70 °C for 40 min. Then the filtrate was concentrated to 1/4 of the original volume after the filtration. Then the solution was extracted with petroleum ether to remove the fat-soluble impurities before and lyophilized to get the final crude RPEFs. The content was determined by the establishment of standard curve (y = 0.09831x-0.00475, R^2^ = 0.9992) in Fig. S1, which y is rutin absorbance and × is concentration of rutin solution (Jia et al., 1999).

### Resin screening

A conical flask (100 mL) contained the pretreated resins (2.00 g) and 2.0 mg/mL flavonoid solution (50 mL), which were placed in an oscillator at 160 rpm and reacted at room temperature for 12 h. After the filtration, the resins were fully transferred into deionized water environment. The desorption process was employed with 50 mL 95 % ethanol solution to desorb the RPEFs in the same system at 160 rpm for 12 h at ambient. The adsorption/desorption amount, and desorption ratio were calculated by Formulas (1), (2), (3).

(1) $$Q_{e}=\frac{\left(C-C_{e}\right)V_{\text{e}}}{m}$$

(2) $$Q_{d}=\frac{C_{d}V_{d}}{m}$$

(3) $$D\left(\%\right)=\frac{C_{d}V_{d}}{\left(C-C_{e}\right)V_{\text{e}}}\times 100\text{\textbackslash{}\%}\;$$

Q_e_ and Q_d_ are equilibrium adsorption and desorption (mg/g resin). C means initial concentration (mg/mL). C_e_ refers to equilibrium concentration (mg/mL). C_d_ was concentration of flavonoids in the desorption solution (mg/mL). m represents resin weight (g). V_e_ and V_d_ mean volume of original sample and desorption solution (mL), respectively. D represents desorption rate (%).

### Static adsorption and desorption experiments

#### Adsorption isotherms

Eight portions of 2.00 g selected resin were prepared into each conical flask, in which 50 mL aqueous solution of RPEFs with the initial concentrations of 0.4, 0.8, 1.2, 1.6, 2.0, 2.4, 2.8, and 3.2 mg/mL were loaded in a thermostatic oscillator respectively. The adsorption behavior was conducted under 25 °C, 35 °C and 45 °C with 250 r/min oscillation for 12 h to collect the adsorption data of RPEFs on the selected resin, which was curved based on the collection data of C_e_ and Q_e_.

Adsorption equilibrium refers to the relationship between the equilibrium concentration of the adsorbent and the free solute concentration in the solution, which can be acquired by the fitting of the adsorption isotherm curve (Kinniburgh, 1986). In this study, three classical isotherm equations including Langmuir, Freundlich and Temkin models were performed to evaluate the adsorption isotherm process. Formula (4), (5), (6) represent the Freundlich, Langmuir, and Temkin models respectively as follows:

(4) $$\mathrm{lg}Q_{e}=\frac{1}{n}\mathrm{lg}C_{e}+\mathrm{lg}K_{F}$$

(5) $$\frac{C_{e}}{Q_{e}}=\frac{1}{K_{L}Q_{m}}+\frac{C_{e}}{Q_{m}}$$

(6) $$Q_{\text{e}}\;\text{=}\;B_{T}\ln C_{e}+B_{T}\ln K_{T}$$

Where Q_e_ is the adsorption capacity (mg/g resin); C_e_ represents the equilibrium concentration (mg/mL); K_F_ and n are empirical coefficients of the Freundlich equation, 1/n represents the intensity of adsorption; Q_m_ is the saturated adsorption capacity (mg/g resin), K_L_ refers to the Langmuir theoretical adsorption coefficient; K_T_ and B_T_ represent constants of the Temkin model.

#### Adsorption kinetics

The initial concentration of RPEFs solution was set as 2 mg/mL for the study of the kinetic adsorption. 2.0 g selected resin were added into 100 mL container and then kept under a constant temperature oscillator (220 r/min) at room temperature. The RPEFs samples of different concentration were executed at time interval of 30, 60, 90, 120, 150, 180, 210, 240, 270, 300, 360, 420, 480, 540, 600, 660 and 720 min, respectively. The adsorption kinetics curve was plotted with time (t) as the abscissa and the adsorption amount (Q_t_) at three levels of temperatures as the ordinate.

In order to get the kinetic adsorption property of RPEFs on the selected resin, the tested data were judged by pseudo-first-order, pseudo-second-order and Weber-Morris intra-particle diffusion models depicted in formulas (8), (9), (10). The adsorption capacity at the corresponding time was analyzed by using Formula (7).

(7) $$Q_{t}=\frac{\left(C_{0}-C_{t}\right)V}{m}$$

(8) $$\ln\left(Q_{\text{1}}-Q_{t}\right)=\ln Q_{\text{1}}-K_{1}t$$

(9) $$\frac{t}{Q_{t}}=\frac{1}{K_{2}Q_{\text{2}}^{2}}+\frac{t}{Q_{\text{2}}},\nu_{0}=K_{2}Q_{2}^{2}$$

(10) $$Q_{t}=K_{w}t^{1/2}+C$$

Where Q_t_ is the cumulative adsorption amount of RPEFs on the resin at any time t (min) (mg/g resin); Q_1_ and Q_2_ are the theoretical adsorption amount at equilibrium (mg/g resin); C_0_ and C_t_ refer to the initial concentration (mg/mL) and the concentration of RPEFs at t time (mg/mL); V represents the volume of the solution (mL); m is the resin weight (g). K_1_, K_2_ and K_w_ mean the rate constants for pseudo-first-order (min^−1^), pseudo-second-order [g/(m·min)] and Weber-Morris diffusion [mg/(g·min^0.5^)], respectively; ν_0_ is the initial adsorption rate (mg/g·min), C is the constant of the diffusion model (mg/g).

### Dynamic adsorption and desorption tests

Dynamic interaction of RPEFs was employed on a 60 mL (1-bed volume (BV)) chromatographic column (20 × 400 mm) filled with the pretreated resin in a wet loading column. At the flow rate of 1, 2, 3, and 4 BV/h, 2 mg/mL RPEFs solution was loaded onto the column packed with the selected resin for the adsorption test. A portion of the solution was collected every 30 mL, and the flavonoids content of each solution was determined to plot the dynamic adsorption breakthrough curve. Dynamic desorption process was under a fixed flow rate of 2 BV/h to load distilled water and serials concentration of ethanol (10 %-90 %) onto the sample-loaded column. Subsequently, the dynamic desorption curve was obtained by desorbing the adsorption bed at 1, 2, 3 and 4 BV/h under the optimal ethanol solution. The recovery rate of flavonoids was calculated by formula (11).

(11) $$R\left(\%\right)=\frac{C_{d}V_{d}}{C_{0}V}\times 100\%$$

C_d_ is the concentration of RPEFs in desorption solution (mg/mL); V_d_ means the desorption solution volume (mL); R is the recovery (%).

### UPLC-Triple-TOF-MS/MS analysis of bamboo shoots residues extracts after enrichment

The purified RPEFs were quantitatively analyzed by using ACQUITY UPLC HSS T_3_ (50 mm × 3.0 mm, 1.8 μm, Waters Technologies Co., Massachusetts, USA) coupled with Triple-TOF 5600^+^ Mass Spectrometry (AB SCIEX CO., Framingham, USA). 0.1 % formic acid solution (A) and acetonitrile (B) were adopted as the mobile phase. The extract was filtered through 0.45 μm membrane prior to 4 μL of injection into the system. The flow rate was maintained at 0.5 mL/min and detected at 280 nm UV length. The elution procedure was presented as following: 0–10 min, 5 % to 40 % B; 10–17 min, 40 %-95 % B; 17–25 min, 95 %-5% B. The MS conditions was set as positive/negative ion sweeping mode: scan range 100–1500 *m*/*z*, ion source gas 1 and 2 (air) 55 psi, curtain gas (N_2_) 35 psi, ion source temperature 600℃ (positive) and 550℃ (negative), source voltage −5.5 kv (positive) and −4.5 kv (negative), collision energy spread 40 ± 20 eV. Data was processed using Analyst software version 4.1 (MassLynx).

### Study on hypoglycemic activity of enriched RPEFs *in vitro*

#### Establishment of insulin resistance model in HepG2 cells

HepG2 cells (human liver cancer cells) collected from the Chinese Academy of Sciences were cultured in DMEM containing 10 % fetal bovine serum. The density of HepG2 cells was adjusted to 5 × 10^4^ cells/mL and inoculated in 96-well plates at 200 μL/well. After 24 h, the culture nutrient of the model group was replaced with an insulin culture medium (insulin was dissolved in 5 % FBS medium), and the control group was cultured normally (containing 5 % FBS). Then the insulin resistance model (IR) was established after another 24 h. To test the stability of the model, the glucose consumption of each group was detected every 24 h using glucose oxidase method.

#### Glucose consumption assay

For the investigation of effect of the enriched RPEFs on HepG2 cells exposed to high concentration (15 μg/mL) of insulin, the experiments were designed into normal group (Control), IR model group and sample groups. According to the Cell Counting Kit-8 method, the cells were cultured in DMEM containing 15 μg/mL insulin for 24 h. Then, the Control, IR, metformin-positive control group (MET, 100 μg/mL) and three different concentrations of RPEFs (200, 400, 800 μg/mL) were replaced and continually cultured until the addition of CCK-8 solution after 24 h. The plates were bred for 1 h incubation and measured at 450 nm to obtain cell activity. Glucose consumption was measured by the absorbance detection of each well at 505 nm by Multiscan MK3 automatic microplate reader (Thermo Lab Systems, USA). Each experiment was repeated three times.

(12) $$C_{g}=\frac{A_{\mathrm{sp}}}{A_{s}}\times C_{s}$$

Where C_g_ is the glucose concentration (mmol/L), A_sp_ refers to the sample absorbance, A_s_ is the calibrator absorbance, and C_s_ means the concentration of the calibrator (mmol/L).

#### Western blot analysis

The procedure was performed following the previously described reference with some modifications (Liu et al., 2021). Originally, HepG2 cells were cultured in a petri dish (60 mm × 15 mm) treated with the control group, IR model group, MET group and different concentrations of RPEFs groups. After washing with precooled PBS three times, each well was ice-cracked with 150 μL RIPA lysis buffer and centrifuged for the determination of protein concentration by a BCA kit. Thereafter, 30 µg of protein were electrophoresed and printed to PVDF membrane (BioTrace™, Pall Corporation, NC, USA). 5 % skim milk powder was used for sealing, and the closed membrane was fully shaken with TBST. After dark incubation at 4 °C overnight, the system was added with the diluted main target antibodies (anti-β-actin (1:1000), anti-Akt (1:1000), anti-PI3K (1:1000), anti-IRS-1 (1:1000), and anti-GLUT4 (1:1000)). Subsequently, the NC membranes were incubated with the corresponding secondary antibody (1:1000) in dark for 2 h and treated with ECL reagent. Finally, the protein band diagram was obtained by fluorescence chemiluminescence gel imaging system, which was carried out by Image Lab software.

### Data analysis

All data were presented as mean ± standard deviation (SD) of three replicates. Statistical differences among groups were analyzed by Student's *t*-test. The p value below 0.05 was considered to be significantly different.

## Results and discussion

### Results of resin screening

**Fig. 1:**
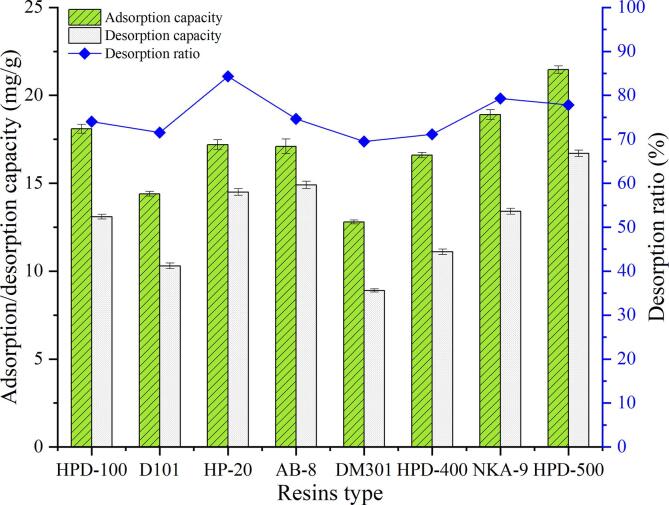
Adsorption/desorption capacity and desorption ratio of total flavonoids of eight resins.

The adsorption/desorption properties of eight resins were studied to find a proper resin for RPEFs, and the results were shown in Fig. 1. The data showed that the adsorption capacities of three resins including HPD-500, NKA-9 and HPD-100 towards RPEFs were higher than those of other resins, and the former had the strongest adsorption capacity, but their desorption capacities and desorption rates were significantly different. Concerning the intrinsic properties of these macroporous materials in Table S1, it was speculated that the accessible surface area would benefit their strong adsorption capacity on RPEFs, in which the values for both HPD-500 and HPD-100 were above 500 m^2^/g. The larger the definite surface area, the more the binding sites of the resin, and the usual adsorption capacity would also increase (Jiang et al., 2020).

The strong polarity might be attributed to the relatively higher adsorption capacity of HPD-500 and NKA-9 resins as well. Flavonoids in bamboo shoots are composed of polar polyhydroxy (Ismail et al., 2020), which may cause a great influence on the adsorption process due to van der Waals force and hydrogen bond. For the analysis of desorption process, three resins including HP-20, AB-8 and HPD-500 had the highest desorption rate compared with others. It revealed that the larger pore size would be helpful for the desorption process. This finding was consistent with the results reported by Hou et al (2020) and was explained by the longer settling time of the eluent on the target components in that scenario (Zou et al., 2015). From the results of adsorption and desorption experiments, it was suggested that HPD-500 resin had both better adsorption and higher desorption capacity for the purification of RPEFs than other resins. Therefore, this macroporous resin was selected to enrich flavonoids from bamboo shoots residues followed by the optimization of related operating conditions.

### Adsorption isotherms

**Fig. 2:**
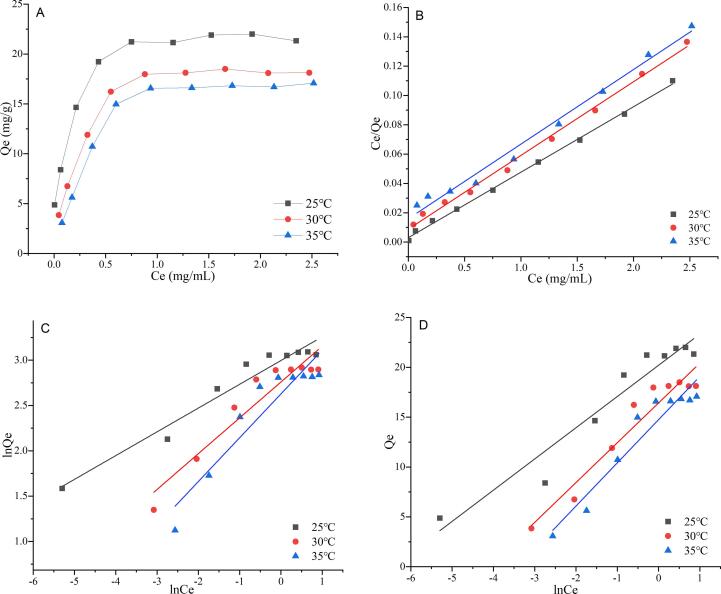
Adsorption isotherms (A) and linear correlations on the basis of the Langmuir (B), Freundlich (C) and Temkin (D) models for RPEFs on HPD-500 resin at 25, 30 and 35℃. Qe refers to equilibrium adsorption capacity and Ce means equilibrium concentration.

It's of great significance to study the equilibrium adsorption isotherms, which would be attributable to the understanding of interaction between flavonoids and resins and optimization of operation parameters. In this test, the adsorption isotherms of RPEFs on HPD-500 resin were implemented at 25, 35 and 45 °C in Fig. 2. It was notable that the trend of adsorption onto HPD-500 resin declined obviously with the increase of temperature, which uncovered the combination of RPEFs with the resin was exothermic. At every stage of the temperature, the adsorption behavior of HPD-500 on RPEFs climbed remarkably within 1 mg/mL and then rose slowly to reach the highest point from the starting point of 2 mg/mL.

The Langmuir model describes the process adheres to monolayer adsorption, in that scenario there are no significant difference of energy in the adsorption sites and no interaction among the adsorbed molecules (Wu et al., 2016). However, in the Freundlich model, the adsorption sites are assumed to be located on the surface with different energy on the heterogeneous phase. This indicates that both monolayer and multilayer adsorption can be fit well by the Freundlich model (Yang et al., 2012). The Temkin model is to consider the interaction between the adsorbent and the adsorbed substance in a linear mode (Khenifi et al., 2010). The fitting results applied with these empirical models were shown in Fig. 2 and Table S2 provided the isotherms equations and the related parameters. Considering the R^2^ value (0.9882 ∼ 0.9980), the Langmuir model at different temperatures was more appropriate for the adsorption fitting of RPEFs on HPD-500 resin in comparison to other two models, which confirmed the total flavonoids from bamboo shoot residues were more inclined to monolayer adsorption on the selected resin. This phenomenon has also been reported in the model for total flavonoids from kale (Chen et al., 2022). Hou et al. (2020) also found the adsorption of total flavonoids from *Pteris ensiformis* on NKA-II resin was a monolayer model. In the Freundlich model, n can represent the adsorption strength and the values were greater than 1 at three temperatures, indicating that HPD-500 polar resin had good adsorption capacity for RPEFs. While the correlation coefficients of the Temkin model were greater than 0.9, suggesting that HPD-500 resin adsorption of RPEFs might be mixed with some multilayer adsorption (Foo & Hamed, 2010). Moreover, the values of K_T_ were sharply smaller from 633.37 to 30.07 L/mg within the tested temperature range, which evidenced that higher temperature was unfavorable to adsorption (Hou et al., 2020). Consequently, the results recommended 25℃ was selected for the adsorption of RPEFs towards HPD-500 resin for large-scale industrial production with relatively lower costs and energy at 2.0 mg/mL.

#### Adsorption dynamics

**Fig. 3:**
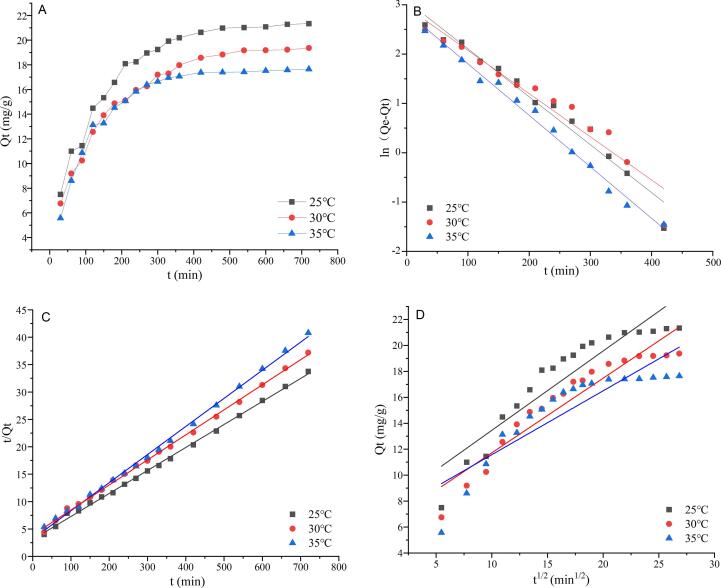
Adsorption kinetic curves (A) and linear correlations on the basis of the pseudo-first-order (B), pseudo-second-order (C) and Weber-Morris (D) models for RPEFs on HPD-500 resin at 25, 30 and 35℃. Qt represents the cumulative adsorption amount of RPEFs on the resin.

The mechanism of dynamic adsorption can be obtained by the rate control and data fitting of the adsorption process (Hou et al., 2019). In this study, three classic mathematic models were carried out to investigate the principle of HPD-500 polar resin for RPEFs, ie. Pseudo-first-order, Pseudo-second-order and Weber-Morris intra-particle diffusion models. As shown in Fig. 3, the adsorption capacity towards RPEFs subjected to HPD-500 resin climbed rapidly in the initial 200 min, and then gradually grew up until the equilibrium was approached at 480 min. The reason could be explained that more fresh binding sites of macroporous resin would be provided for the flavonoids during the initial process of adsorption. Then the adsorption rate tended to be slow down due to the saturation of binding positions and narrowness of gap between the adsorbates and adsorbents. This trend was as same as that of chlorogenic acid combined with NKA-Ⅱ resin (Jiang et al., 2020).

Different from the pseudo-first-order model regarding chemical adsorption as a rate-limiting step, the pseudo-first-order model is generally applied to fit the whole adsorption data. The Weber-Morris intra-particle diffusion model is mainly conducted to prove whether there is intraparticle diffusion during the adsorption process (Chen et al., 2016). The relevant parameters of adsorption kinetics equations were established to study the adsorption rate and reveal the potential mechanism in Table S3. At three temperatures, the R^2^ values of the pseudo-second-order model were 0.9983, 0.9984, and 0.9980, respectively, higher than those of other two models. Moreover, there was a close correlation and consistency between the actual equilibrium adsorption Q_e_ value (21.23 mg/g) of the pseudo-second-order model and the theoretical equilibrium adsorption Q value (21.74 mg/g) at 25℃. In the Weber-Morris model, the linear fitting plots at different temperatures did not coincide with the origin, suggesting both the internal and external diffusions of particles happened in the main control parts of the adsorption rate (Noroozi et al., 2007, He et al., 2013). Consequently, the pseudo-second-order may be an appropriate model to stimulate the kinetic adsorption for RPEFs on HPD-500 resin (Chen et al., 2022). Jiang et al. (2020) reported this kind of model was suitable for the analysis of the whole adsorption process as the rate-limiting step.

### Selection of elution concentration

**Fig. 4:**
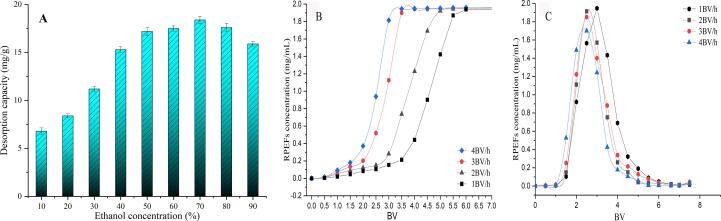
Effect of ethanol concentration on desorption capacity of HPD-500 resin (A); Dynamic adsorption breakthrough curve (B) and Dynamic desorption curve (C).

The desorption of RPEFs on HPD-500 resin depends on the adsorption competition between intermolecular forces on resin and solvents. When intermolecular force decreases, RPEFs is easily desorbed from HPD-500 resin to solvent (Chang et al., 2012). Thus, the eluent concentration is crucial for desorption process to separate the sample from the adsorbed resin. Fig. 4A depicted that the desorption capacity of HPD-500 went up rapidly and hit the top of 18.16 mg/g at 70 % ethanol concentration within a range of ethanol concentration (10 % to 50 %). Continuous rise of the elution concentration from 70 % to 90 %, the capacity decreased slightly. The reason might be attributed to the polarity of solvent. The higher concentration of ethanol was, the lower polarity of system. The results showed that 70 % ethanol with relative moderate polarity had a stronger ability to reduce the affinity between RPEFs and HPD-500 resin, which was conducive to more efficient desorption.

### Dynamic test results

#### Dynamic penetration curve of HPD-500 resin

The dynamic penetration curve was acquired by measuring the concentration of flavonoids in the different flow rates through the chromatographic column. It’s commonly known that one-tenth of the initial concentration in solution is set as the breakthrough point (Zhao et al., 2015). In this experiment, the value would be captured once the concentration of the collection solution reach 0.2 mg/mL. Fig. 4B depicted the dynamic breakthrough curves at different rates from 1 BV/h to 4 BV/h on HPD-500 resin. The slower the flow rate of eluent, the greater the adsorption capacity of resin. Unfortunately, those facilitated an interaction between the adsorption molecule and the active site of HPD-500 resin, leading to a longer time to reach the warning point. At flow rate of 1, 2, 3 and 4 BV/h, the leak volumes were 3.5, 2.8, 2.1 and 1.64 BV, respectively. Taking the adsorption capacity and efficiency into account, 2 BV/h of flow rate and 2.8 BV of treatment volume were considered to be the best conditions for dynamic adsorption.

#### Dynamic desorption curve of HPD-500 resin

The desorption curves were obtained by eluting the adsorbed column with 6 BV distilled water and 70 % ethanol at 1, 2, 3 and 4 BV/h, respectively (Fig. 4C). It was remarkable that the desorption curves presented narrow and sharp peak profiles at the flow rate of 2 and 3 BV/h. The eluent volume postponed with the decrease of flow rate. Although a low flow rate was beneficial to the interaction between desorption solution and flavonoids (Sandhu & Gu, 2013; Jiang et al., 2020), time increment was not conducive to industrial application. Compared with 3 BV/h (77.65 % of flavonoids recovery), the desorption efficiency and flavonoids recovery under 2 BV/h was relatively superior (83.21 % of flavonoids recovery). Hence 2 BV/h flow rate was adopted for efficient desorption. Under this optimized process, 70 % desorbed solution was collected by freeze-dry. And the final content of flavonoids in RPEFs after the enrichment by HPD-500 resin was 37.34 %, which was memorably increased from 1.39 % in the crude RPEFs.

### Monomer composition of RPEFs

**Fig. 5:**
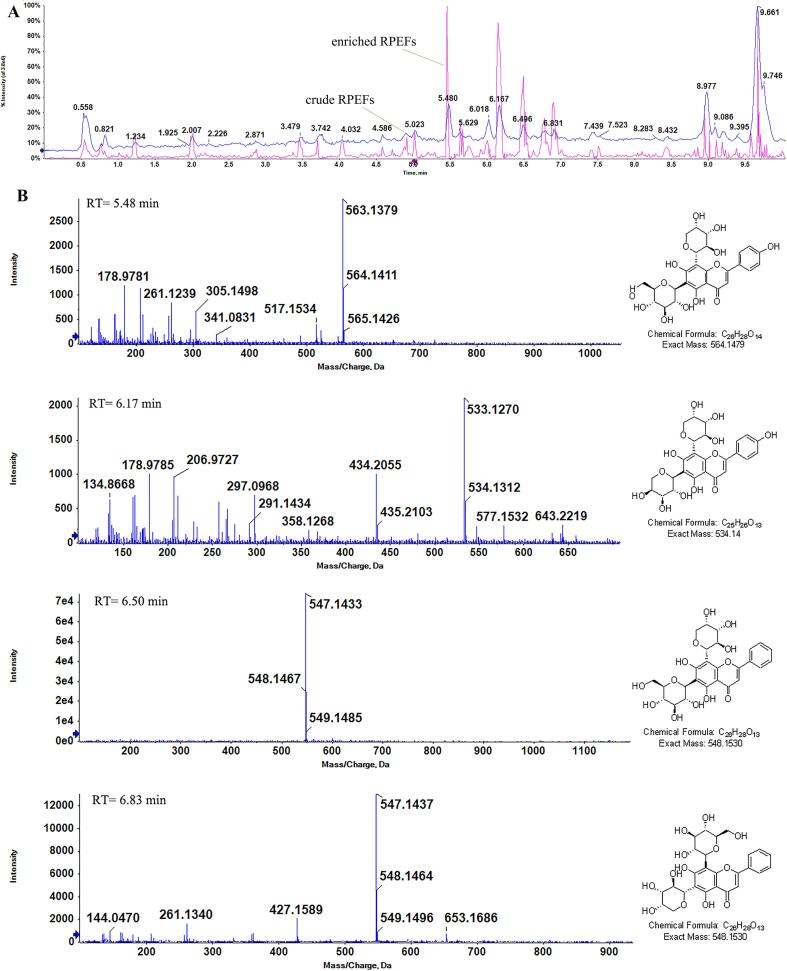
The UPLC chromatograms of RPEFs detected at 280 nm (A) and mass spectra of four RPEFs glycosides (B). a-1, b-1, c-1, d-1 represent the mass spectra of compounds a, b, c, d in turn; a-2, b-2, c-2 and d-2 refer to the chemical formula of a, b, c and d, respectively.

The physicochemical analysis of the enriched RPEFs were employed with UPLC-Triple-TOF/MS and the total ion flow diagram was obtained in Fig. 5A. It was obvious that the contents of RPEFs were significantly increased after the purification by HPD-500 resin, especially some bioactive compounds ranging from retention time (RT) 5.0 to 7.0 min in total ion flow profiles. Normally structural information of flavonoids can be acquired by identifying the ion fragments patterns after cleavage with published data and measuring the retention time and empirical formula. In this elucidation of their retention time and mass spectra, four major compounds (flavonoid glycosides) were presented with tentative chemical formula in Fig. 5B, which were Apigenin 8-C-α-l-arabinoside 6-C-β-d-glucoside (schaftoside, RT 5.48 min), Apigenin 6-C-α-l-arabinopyranoside-8-C-β-d-xylopyranoside (RT 6.17 min), Chrysin 6-C- arabinoside 8-C-glucoside (RT 6.50 min) and Chrysin 6-C-glucoside 8-C-arabinoside (RT 6.83 min), and the total flavonoids content was 34.16 %, which was close to the above results of eluted components determined by using the NaNO_2_-Al(NO_3_)_3_-NaOH colorimetric method.

### Study on hypoglycemic activity

#### Effects of RPEFs on the activity and glucose consumption of HepG2-IR cells

**Fig. 6:**
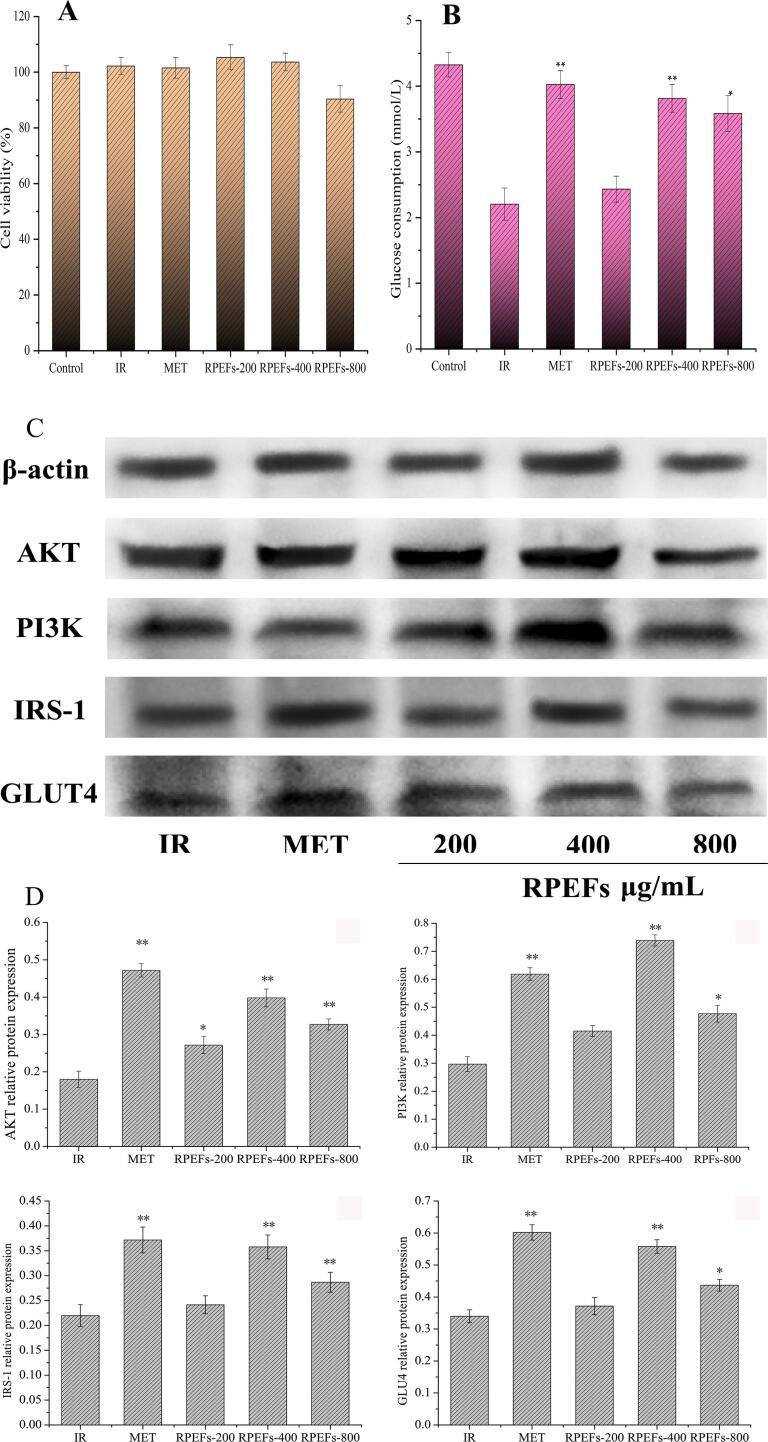
Effect of different concentrations of RPEFs on the activity of HepG2-IR cells (A) and glucose consumption of HepG2-IR cells (B); Western blot analysis of expression levels of AKT, PI3K, IRS-1 and GLUT4 proteins in HepG2-IR cells after treatment by different concentrations of RPEFs for 24 h (C); Quantitative analysis of relative expression of AKT, PI3K, IRS-1 and GLUT4 proteins normalized to β-actin (D). IR: model group, MET: metformin group, others were RPEFS at different concentrations group. Data are expressed as mean ± SD of three independent experiments, *p < 0.05, **p < 0.01.

The activity of HepG2 cells treated with RPEFs at different concentrations was observed in Fig. 6A. The results showed when the RPEFs concentrations were 200 and 400 μg/mL, the activities of HepG2 cells were not significantly inhibited until its concentration reached 800 μg/mL, the value decreased by 10 % compared to the control. Fig. 6B depicted the glucose consumption of the RPEFs group was superior to that of the IR negative control group although the value was not higher compared with that of MET positive group. At 400 μg/mL of RPEFs, the consumption rate increased most significantly (P < 0.01), and the glucose consumption reached 3.56 ± 0.3 mmol/L, which was nearly as twice as that of IR negative control group. Despite the rate slightly decreased at 800 μg/mL, it was still remarkably bigger than that of IR group with 2.36 ± 0.27 mmol/L of glucose consumption. Those results indicated that RPEFs at different concentrations could promote the uptake and utilization of glucose by IR cells. Concerning the weak inhibition of cell activity against HepG2 cells at relatively higher treatment of 800 μg/mL, the optimum hypoglycemic activity on HepG2 cells exposed by RPEFs would be achieved at 400 μg/mL. Zhang et al (2015) reported that flavonoids-rich Chinese bayberry pulp extracts had potent improvement on the glucose consumption *in vitro* due to their five flavonoids inside. Likewise, there were four major flavonoids identified in the purified RPEFs, which might be strongly attributable to their potent hypoglycemic activity.

#### The expression level of related proteins

It has been evidenced that PI3K/AKT and MAPK are involved into insulin signal transduction pathways, which are dependent on activation of the insulin receptor by phosphorylation and recruitment of series of downstream signaling molecules. In this study, the possible hypoglycemic mechanism of RPEFs was predicted by *in vitro* study of the PI3K/AKT pathway of HepG2-IR cells. PI3K, known as phosphatidylinositol 3 kinase, affects cell growth, proliferation and glucose transport. Originally, AKT (a serine/threonine kinase) in the upstream regulator can be triggered by the phosphorylation of PI3K. After insulin activation, its receptor InsR would be activated to form IRS-1 by phosphorylation. Correspondingly, downstream signaling GLUT4 starts to transport glucose into cells by metabolic regulation of glucose and lipid during the glucose consumption of the treated HepG2-IR cells. From the results of western blotting in Fig. 6C-D, it revealed that concentration-dependent RPEFs had a significantly up-regulated effect on the protein expression of PI3K, AKT, IRS-1 and GLUT4 in the PI3K/AKT pathway. And it was intriguingly evidenced that the regulatory effect on the IRS-1/PI3K/Akt/GLUT4 pathway was even slightly stronger than that of MET treatment at 400 μg/mL. This finding further evidenced that flavonoids-rich RPEFs was capable of significant decreasing blood glucose levels and improving tolerance *in vitro* at certain concentration, which was consistent with the study reported by Cheng et al (2020).

## Conclusions

The purification process of total flavonoids from bamboo shoots residues of *Phyllostachys edulis* was investigated by studying the adsorption/desorption of RPEFs on the selected resin in this research. Among the eight conducted microporous resins, HPD-500 resin had the best satisfactory adsorption/desorption performance and recovery. The analysis of adsorption isotherm and kinetic data indicated the Langmuir equation and the pseudo-second-order model can accurately describe its static adsorption on HPD-500 resin. The optimum purified process was as following: 2 mg/mL of flavonoids solution, 2.8 BV feed volume under 2 BV/h flow rate on HPD-500 resin for adsorption; 5 BV volume of 70 % ethanol with a flow rate of 2 BV/h were needed to completely desorb RPEFs. After the enrichment by HPD-500 polar resin, the content of flavonoids in the extract improved from 1.39 % to 37.34 % with flavonoids recovery of 83.21 %. The results of UPLC-TOF-MS/MS uncovered that the purified RPEFs was composed of four major flavonoids, which were Apigenin 8-C-α-l-arabinoside 6-C-β-d-glucoside (schaftoside), Apigenin 6-C-α-l-arabinopyranoside-8-C-β-d-xylopyranoside, Chrysin 6-C-arabinoside 8-C-glucoside and Chrysin 6-C-glucoside 8-C-arabinoside. The results of hypoglycemic activity indicated that concentration-dependent RPEFs could significantly upregulate the protein expression of PI3K, AKT, IRS-1 and GLUT4 in the PI3K/AKT pathway based on the HepG2-IR cells. In conclusion, RPEFs from the bamboo shoot residues of *Phyllostachys edulis* could be purified by HPD-500 macroporous resin and the enriched ones would be valuable for their industrial application.

## Declaration of Competing Interest

The authors declare the following financial interests/personal relationships which may be considered as potential competing interests: Liang He reports financial support was provided by Forestry Department of Zhejiang Province. Liang He reports financial support was provided by Zhejiang provincial science and Technology Department.

## Data Availability

The research data are available on line.
