# Supplementary material for: Efficient purification of flavonoids from bamboo shoot residues of Phyllostachys edulis by macroporous resin and their hypoglycemic activity
Source: Food Chem X. 2022 Nov 12;16:100505. doi: 10.1016/j.fochx.2022.100505 (PMC9743288; doi:10.1016/j.fochx.2022.100505)
Supplement: Supplementary data 1 [file mmc1.docx]

Efficient purification of flavonoids from bamboo shoot residues of *Phyllostachys edulis* by macroporous resin and their hypoglycemic activity

Yanbin Wang^a^, Yalan Zhang^a,c^, Junwen Cheng^a^, Jiancheng Zhao^b^, Rui Shi^c^, Liang He^a,*^, Qin Li^b,*^ Yongjian Chen^d^

a The Key Laboratory of Biochemical Utilization of Zhejiang Province, Department of Forest Foods, Zhejiang Academy of Forestry, Hangzhou 310023, China

b Bamboo Shoots Engineering Research Center of the State Forestry Bureau, Department of Bamboo, Zhejiang Academy of Forestry, Hangzhou 310023, China

c College of Light Industry and Food Engineering, Nanjing Forestry University, Nanjing 200093, China

d Zhejiang Shengshi Biotechnology Co., Ltd., Huzhou 313399, China

*Corresponding authors. Tel.: +86 571 87798225; Fax: +86 571 87798206.

E-mail address: kite006@126.com (L. He), 18258174992@139.com (Q. Li)


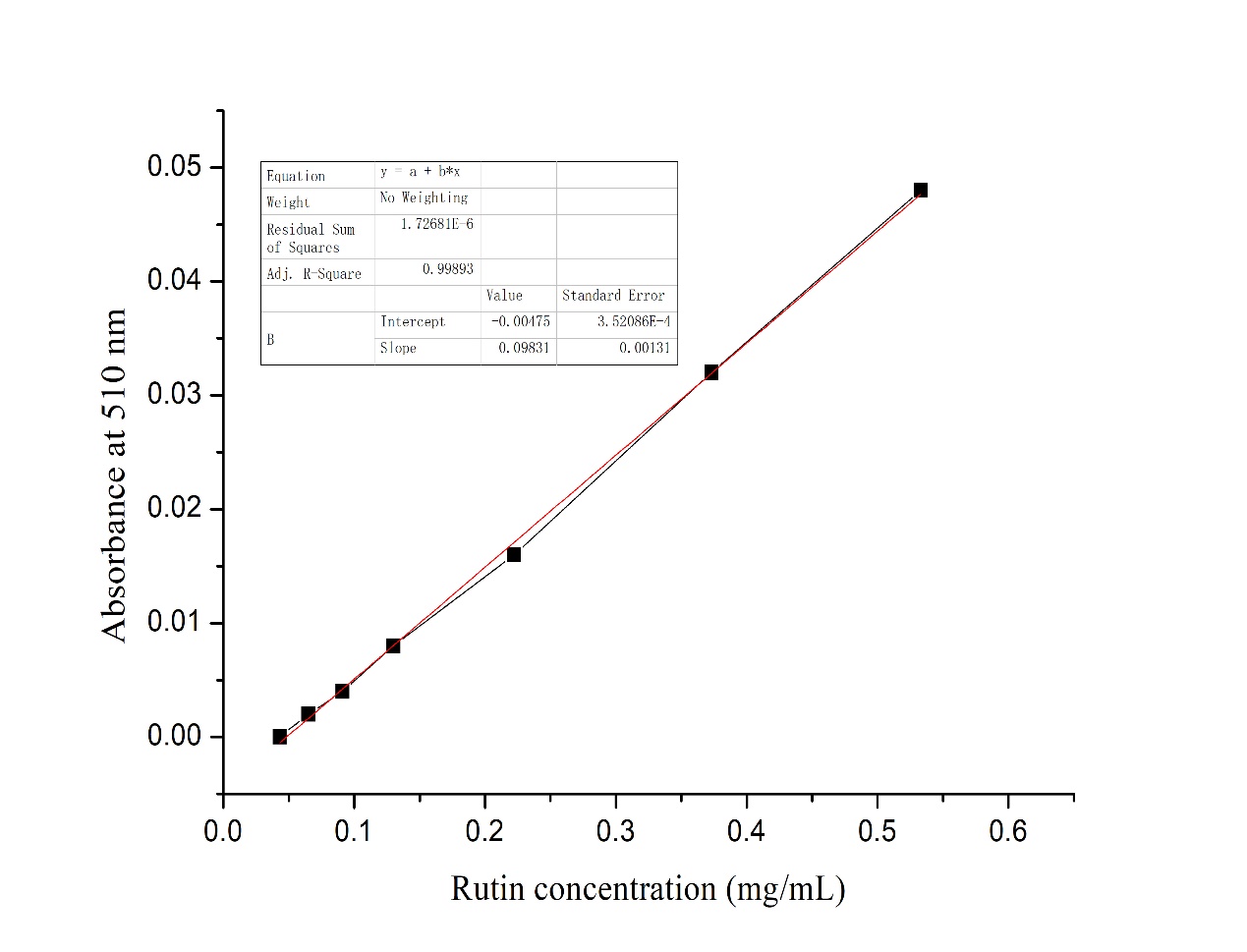


Figure S1 The standard curve of rutin

Table S1 Characteristics of eight macroporous resins

| Resin | Polarity | specific surface area(m^2^/g) | average pore size(Å) |
| --- | --- | --- | --- |
| HPD-100 | non-polar | 650~700 | 85~90 |
| D-101 | non-polar | 500~550 | 90~100 |
| HP-20 | non-polar | 550~600 | 290~300 |
| AB-8 | weak polarity | 480~520 | 130~140 |
| DM-301 | moderately polar | 330~380 | 130~170 |
| HPD-400 | moderately polar | 500~550 | 75~80 |
| NKA-9 | polarity | 250~290 | 155~165 |
| HPD-500 | polarity | 500~550 | 55~75 |

Table S2 The fitting parameters of adsorption isotherms of RPEFs on HPD-500 polar resin at different temperatures

| Temperature(℃） | Freundlich model | | | Langmuir model | | | Temkin model | | |
| --- | --- | --- | --- | --- | --- | --- | --- | --- | --- |
|  | K_F_ | n | R^2^ | K_L_ | Q_m_ | R^2^ | B_T_ | K_T_ | R^2^ |
| 25 | 20.0059 | 3.8142 | 0.9507 | 14.9365 | 22.3914 | 0.9980 | 3.1318 | 633.3673 | 0.9353 |
| 35 | 15.7796 | 2.5304 | 0.9040 | 5.7289 | 19.8807 | 0.9955 | 3.9938 | 61.1390 | 0.9315 |
| 45 | 13.8357 | 2.0691 | 0.8786 | 3.1986 | 19.6502 | 0.9882 | 4.3327 | 30.0744 | 0.9215 |

Table S3 Parameters of adsorption kinetics fitting curve of HPD-500 polar resin for RPEFs adsorption.

| Temperature(℃) | Pseudo-first-order | | | Pseudo-second-order | | | Weber-Morris | | |  |
| --- | --- | --- | --- | --- | --- | --- | --- | --- | --- | --- |
|  | Q_1_  (mg/g) | K_1_  (min^-1^) | R^2^ | Q_2_  (mg/g) | K_2_  (g/mg min) | R^2^ | C | K_W_  (mg/g min^1/2^) | R^2^ | Qe  (mg/g) |
| 25 | 21.7386 | 0.0097 | 0.9668 | 21.8265 | 5.70E-04 | 0.9983 | 7.3430 | 0.6113 | 0.8591 | 21.23 |
| 30 | 19.1672 | 0.0088 | 0.9328 | 19.8771 | 5.37E-04 | 0.9984 | 5.9677 | 0.5763 | 0.8984 | 18.12 |
| 35 | 17.2715 | 0.0105 | 0.9911 | 17.5046 | 8.22E-04 | 0.9980 | 6.6547 | 0.4924 | 0.7887 | 16.62 |
